# Necrotising fasciitis with extensive necrosis caused by *Lactobacillus*: a case report

**DOI:** 10.1186/s12879-024-09291-3

**Published:** 2024-04-22

**Authors:** Jun Nagayama, Takeo Sato, Ishida Takanori, Koga Kouji, Nakamura Mitsunobu

**Affiliations:** 1 Advanced Medical Emergency Department and Critical Care Center, Japanese Red Cross Maebashi Hospital, 389-1, Asakura-machi, 371-0811 Maebashi, Gunma Japan; 2 Center for Community Clinical Education, Jichi Medical University, 3311-1 Yakushiji, 329-0498 Shimotsuke-shi, Tochigi-ken Japan

**Keywords:** Debridement, Laboratory risk indicator for necrotising fasciitis score, *Lactobacillus* spp., Necrotising fasciitis

## Abstract

**Background:**

Necrotising fasciitis (NF) is a life-threatening soft-tissue infection that rapidly destroys the epidermis, subcutaneous tissue, and fascia. Despite their low virulence, *Lactobacillus* spp. can cause NF, and because of its rare incidence, there is limited information about its molecular and clinicopathological characteristics. We report a rare case of NF in a patient with type 2 diabetes mellitus diagnosed on admission and severe obesity due to infection with two types of *Lactobacillus* spp. that manifested in extensive necrosis.

**Case presentation:**

A 48-year-old woman was referred to our hospital with a complaint of difficulty walking due to severe bilateral thigh pain. She presented with mild erythema, swelling, and severe skin pain extending from the pubic region to the groin. The patient was morbidly obese, had renal dysfunction, and had diabetes mellitus diagnosed on admission.; her LRINEC (Laboratory Risk Indicator for Necrotising Fasciitis) score was 9, indicating a high risk of NF. An exploratory surgical incision was made, and NF was diagnosed based on fascial necrosis. Emergent surgical debridement was performed, and cultures of the tissue culture and aspirated fluid/pus revealed two types of *Lactobacillus* spp.: *Lactobacillus salivarius* and *L. iners*. The patient was admitted to the intensive care unit (ICU), where antibiotics were administered and respiratory and circulatory management was performed. Diabetic ketoacidosis was detected, which was treated by controlling the blood glucose level stringently via intravenous insulin infusion. The patient underwent a second debridement on day 11 and a skin suture and skin grafting on day 36. The patient progressed well, was transferred from the ICU to the general ward on day 41, and was discharged unassisted on day 73.

**Conclusions:**

*Lactobacillus* spp. are rarely pathogenic to healthy individuals and can scarcely trigger NF. However, these bacteria can cause rare infections such as NF in immunocompromised individuals, such as those with diabetes and obesity, and an early diagnosis of NF is imperative; surgical intervention may be required for the prevention of extensive necrosis. The LRINEC score may be useful for the early diagnosis of NF, even for less pathogenic bacteria such as *Lactobacillus*.

## Background

Necrotising fasciitis (NF) is a life-threatening soft-tissue infection that rapidly destroys the epidermis, subcutaneous tissues, and fascia [1]. Its occurrence is rare, with approximately 4 per 100,000 people per year, with the condition being most frequently diagnosed in individuals between 50 and 60 years of age, with a higher prevalence in men [2]. Risk factors include diabetes, immunosuppression, malnutrition, advanced age, non-steroidal anti-inflammatory drug (NSAID) use, morbid obesity, liver cirrhosis, alcoholism, chronic renal failure, HIV/AIDS, and underlying malignancy [3]. Notably, patients with diabetes and obese individuals account for 22–59% and 17–31% of all cases, respectively. Its clinical presentation includes rapidly progressive inflammation of the skin and soft tissues, with mortality rates ranging from 30 to 40%. Delayed surgical intervention may worsen the prognosis, underscoring the need for prompt diagnosis and therapeutic intervention. NF is categorised according to the causative organisms into mainly two types: type I, a complex infection involving aerobic and anaerobic organisms (polymicrobial), and type II, caused by a single Gram-positive organism (monomicrobial), such as Group A *Streptococcus* or methicillin-resistant *Staphylococcus aureus* (MRSA) [1, 2]. The most common causative organisms are *Streptococcus pyogenes* and *Staphylococcus epidermidis*, followed by Gram-negative rods(GPR) such as *Klebsiella* spp [4].. Although less common, type III infections caused by *Vibrio vulnificus* have also been reported [1]. *Lactobacillus* spp. has rarely been documented to cause NF [5] and is not known to trigger extensive necrosis because of the low virulence of the organism. In this report, we describe a case of NF in a patient, with type 2 diabetes mellitus diagnosed on admission and severe obesity, which was caused by infection with two types of *Lactobacillus* spp. that manifested in extensive necrosis.

## Case presentation

**Fig. 1:**
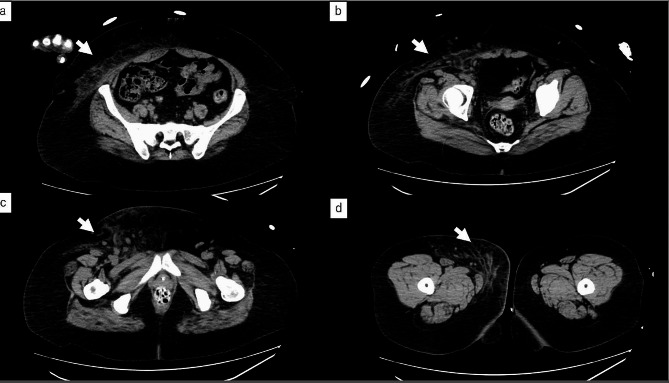
Computed tomography scan of abdominal to femoral region at the first visit. (**a**): lower abdomen, (**b**): pelvic, (**c**): inguinal sutra, (**d**): femoral region, Increased adipose tissue density was observed from the right side of the abdomen to the right thigh (arrows). No gas images were observed

**Fig. 2:**
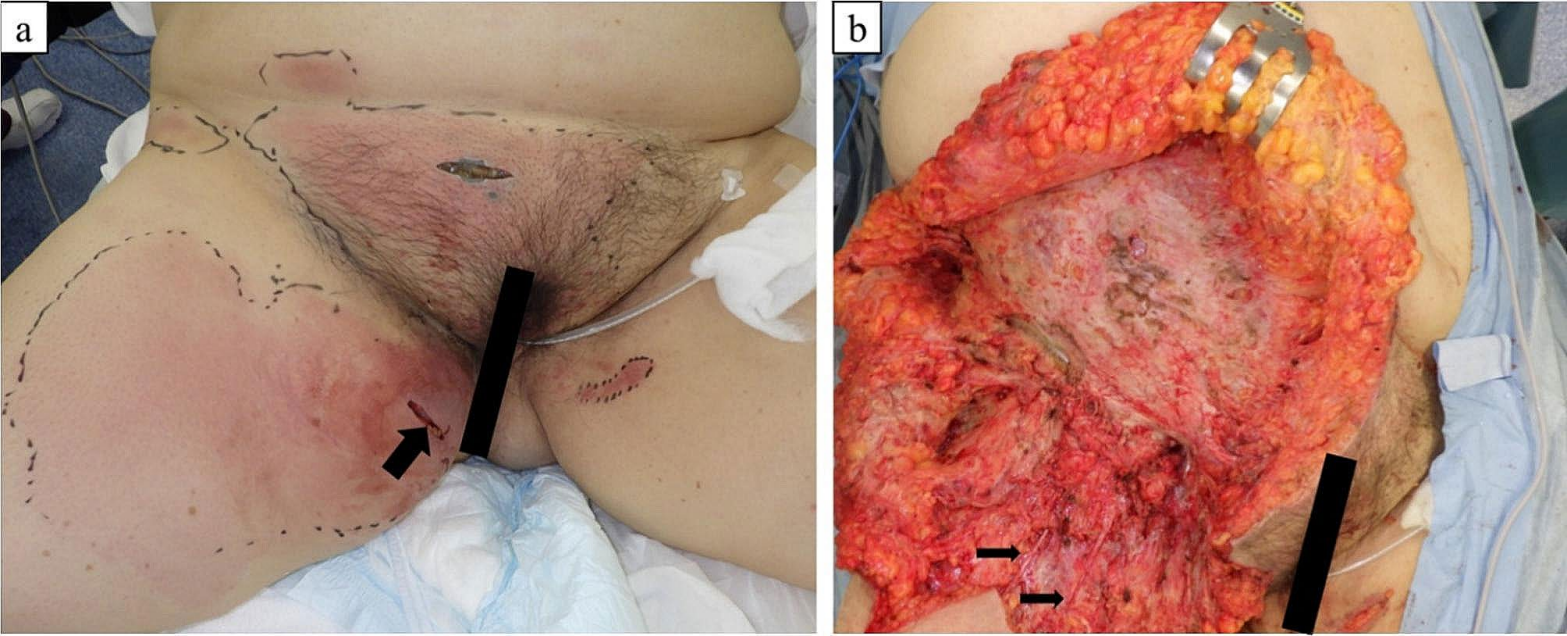
Skin and surgical findings. (**a**) Skin Findings: The skin showed pale erythema and swelling from the pubic to the inguinal region. An area of intense erythema in the medial region of the right thigh, and folliculitis in this area is thought to be the site of entry (arrow). (**b**)Surgical Findings: Extensive necrotic tissue extending from the right lateral abdomen to the anterior aspect of the thigh is observed. Necrosis in the right inguinal region extending to the femoral vein (arrows)

**Fig. 3:**
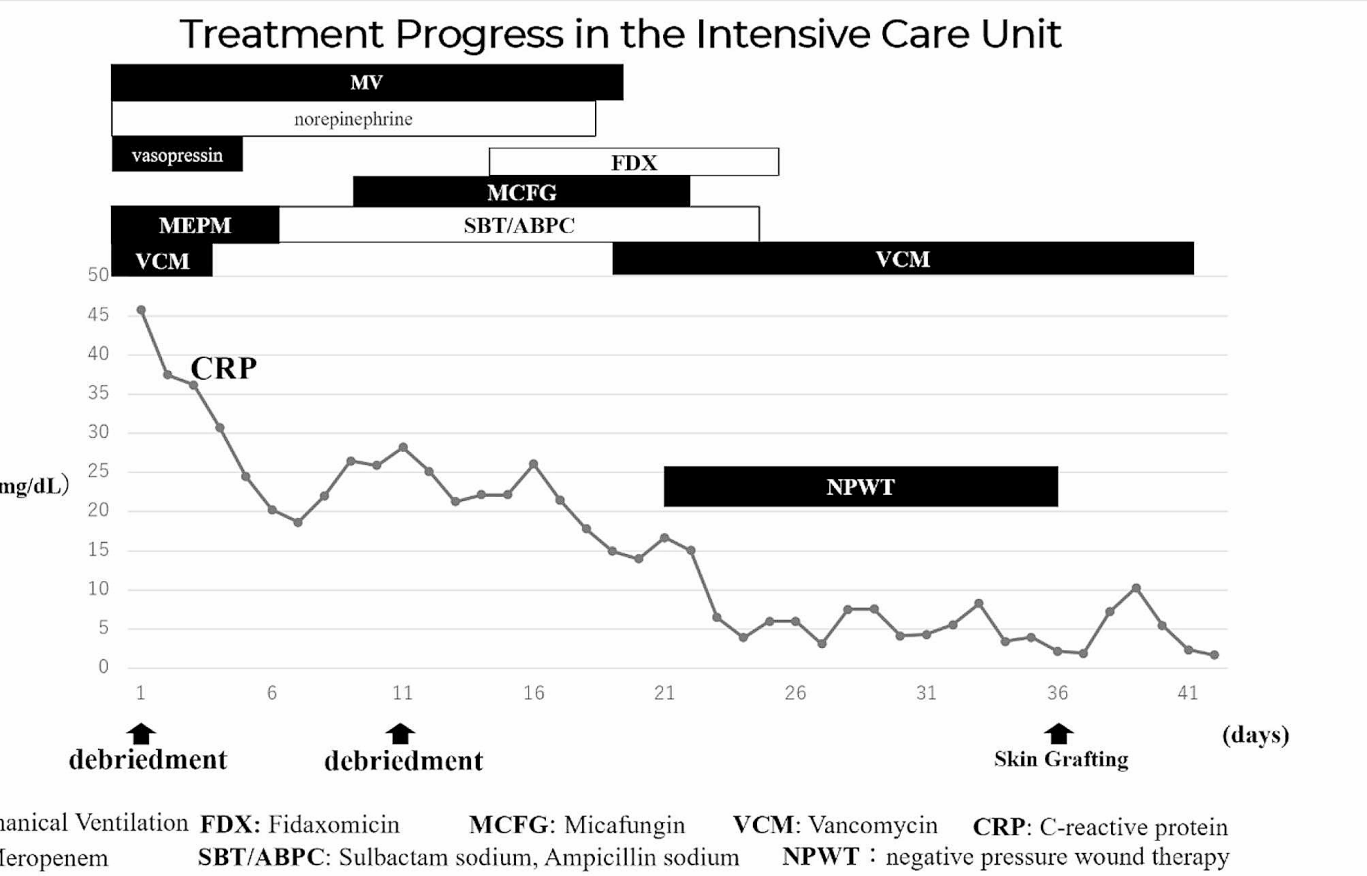
Treatment progress in the intensive care unit (ICU). The figure shows the course of treatment after admission to the ICU. The inflammation improved with debridement and administration of antibiotics. The patient was discharged from the ICU on day 41 after skin grafting

**Fig. 4:**
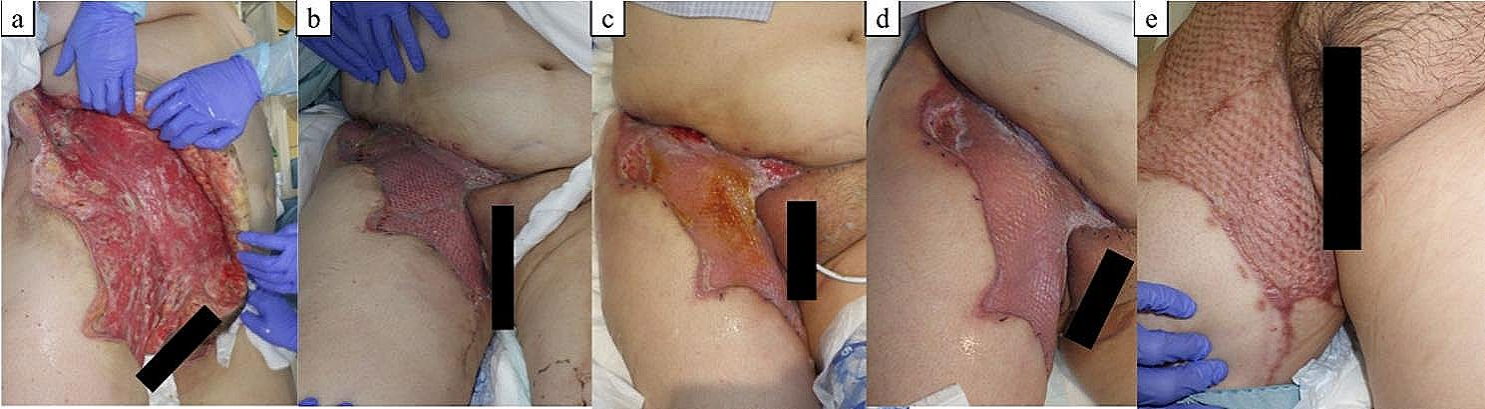
Progression of post-operative skin healing. (**a**): after post-operative day (POD) 19, (**b**): after POD 40, (**c**): after POD 54, (**d**): after POD 68, (**e**): after 3 months. Skin Findings: A skin graft was placed from the right inguinal region to the thigh. The skin grafting was successful

A 48-year-old woman with a history of manic depression and no history of previous NSAID or probiotic use had been treated for bilateral cutaneous candidiasis of the thighs for 3 weeks. The patient visited her family physician because she had experienced severe bilateral pain in her thighs upon waking up in the morning and was unable to walk. Blood tests showed high levels of inflammation (C-reactive protein [CRP] 45.94 mg/dL), and she was referred to our hospital for a detailed examination. Mild erythema and swelling of the skin, extending from the pubic area to the groin, were observed, and she had persistent severe pain in that region. Her height was 158 cm, weight 101 kg, body mass index (BMI) 40.5 kg/m^2^, Glasgow Coma Scale (GCS): E3V5M6, temperature 37.3 °C, pulse 114 beats/min, blood pressure 91/58 mmHg, respiratory rate 21 breaths/min, and oxygen saturation 95% at room air. The patient was severely obese and had impaired consciousness and hypotension. Blood laboratory test values, including those for Laboratory Risk Indicator for Necrotising Fasciitis (LRINEC), were: white blood cell count 12,600/µL (76.5% neutrophils), haemoglobin 14.3 g/dL, platelet count 120,000/µL, Na^+^ 127 mEq/L, creatinine kinase 33 IU/L, lactate 3.0 mmol/L, glucose 741 mg/dL, blood urea nitrogen (BUN) 50 mg/dL, creatinine 1.9 mg/dL, creatinine clearance 57.74 mL/min, CRP 45.71 mg/dL, HbA1c 15.6%, and urine ketone 2 + positive. Type 2 diabetes mellitus was diagnosed at the time of admission. Arterial blood gas analysis showed HCO_3_^−^ of 9.8 mEq/L and an anion gap of 27.6 mEq/L, indicating anion gap-opening metabolic ketoacidosis. Computed tomography showed increased adipose tissue deposition, from the right abdomen to the anterior of the right thigh, with no gas-producing findings (Fig. 1). Initially, cellulitis was suspected; however, since the patient complained of severe pain and was at high risk of NF with a LRINEC score of 9 (4, 2, 2, and 1 points for CRP, sodium, creatinine, and glucose, respectively), an exploratory surgical incision was made, and NF was diagnosed based on the identification of necrotic tissue in the fascia. Emergency debridement surgery was performed on the same day. Intraoperative findings revealed extensive necrotic tissue extending from the right side of the abdomen to the anterior thigh (Fig. 2). The necrosis in the right groin extended to the right femoral vein. The follicle in the medial region of the right femur was the most inflamed and considered the site of bacterial entry, with necrosis more extensive than the skin findings. The patient was admitted to the intensive care unit (ICU). Adequate circulatory status was maintained with extracellular fluid administration, norepinephrine, and vasopressin. The patient required sedation and analgesia during daily wound care. Consequently, ventilatory management was initiated. Tight glycaemic control was maintained with administration of intravenous insulin because of diabetic ketoacidosis. Because the patient had an unstable circulatory status and was receiving high doses of catecholamines, meropenem and vancomycin were empirically administered until the causative bacteria were identified. Gram staining of the tissue culture and aspirated fluid/pus showed an elongated GPR. When specimens were examined for each colony grown on chocolate medium, they revealed two types of *Lactobacillus* spp.: *Lactobacillus salivarius* and *L. iners*. On day 6, report of bacterial susceptibility to antibiotics was obtained; however, no drug resistance was found. Penicillin and ampicillin sodium are first-line antibacterial agents for *Lactobacillus* infections. Therefore, following detection of sensitivity, the antibiotic was changed to sulbactam ampicillin. A second debridement was performed on day 11. *Candida albicans* was detected in the pus from the wound, and was treated with a 9-day micafungin therapy. Pseudomembranous enteritis developed on day 18 and consequently, fidaxomicin was administered for 10 days. The patient was weaned off the ventilator on day 19. Drainage with negative-pressure wound therapy was continued from day 21 to 36. Because MRSA was detected in blood cultures, vancomycin was administered from day 21 to 41. The patient’s condition improved and antibiotics were discontinued on day 25. On day 36, the wound was closed and skin grafted from the right inguinal region to the thigh. The patient was discharged from the ICU on day 41. The skin graft was successful and the patient was discharged without assistance on day 73 (Figs. 3 and 4).

## Discussion and conclusions

We encountered a case of NF caused by *Lactobacillus* spp. The genus *Lactobacillus* comprises anaerobic facultative Gram-positive rods. To date, more than 250 species of this genus have been identified. Common species include *L. rhamnosus* and *L. casein*, which form the natural flora of the oral cavity, gastrointestinal tract, and urogenital tract as commensals [6]. *L. salivarius* and *L. iners*, detected in the present case, are indigenous bacteria in the gastrointestinal tract and vagina, respectively. *Lactobacillus* are lactic acid producing bacteria used frequently in commercial applications, particularly in the fermentation of cheese and other dairy products [7]. The species has probiotic functions including suppression of microbial virulence in the intestinal tract [8]. Under normal physiological conditions, they are less pathogenic and not harmful to healthy individuals. However, there have been reports of the association between the use of probiotics and bacteraemia [9], and the risk of *Lactobacillus* bacteraemia has been reported in patients taking immunosuppressive drugs, as well as those with diabetes and advanced cancer [10].

Severe infections, including endocarditis and meningitis, have been reported to be caused by *Lactobacillus* spp. such as *L. casei*, *L. rhamnosus* and *L. salivarius* [6]. Although less virulent, the mortality rate may be as high as 30% if *Lactobacillus* spp. are pathogenic [6]. *L. rhamnosus* and *L. casei* have potential virulence factors that produce proteins that can bind to extracellular proteins, such as fibrinogen. The ability to bind fibrinogen has also been reported to help Gram-positive pathogens escape the immune system [11]. *L. salivarius* detected in the culture in the present case is also reported to bind to human fibrinogen via a fibrinogen-binding protein and cause aggregation of human platelets; some strains have been found to aggregate human platelets at levels comparable to those of *S. aureus* [12]. These mechanisms may explain the pathogenesis of *Lactobacillus* infections. We found two cases of severe soft-tissue infections caused by *Lactobacillus* spp.in literature: a brief report of a case of NF caused by *L. acidophilus* [5] and another of Fournier’s gangrene caused by *L. acidophilus* described in a review [10]. (Table 1). Notably, all three cases, including the present case, were complicated by diabetes mellitus. Immunity is an important factor in the aetiology of *Lactobacillus* bacteraemia, and 21% of patients with *Lactobacillus* bacteraemia are reported to have diabetes [10], indicating that immunosuppression due to diabetes is an important factor in NF caused by *Lactobacillus*.

**Table 1:** *Lactobacillus*-associated soft-tissue infections

Publication Year	Age (years)	Sex	Clinical symptoms	Diabetes	LB-associated infection	Species	Debridement	Other microorganisms	LRINEC Score	Outcome	Reference
2011	58	M	Sepsis	+	Fournier gangrene	*L. acidophilus*	N/A	*Candida. glabrata*	N/A	Cured	[10]
2018	59	F	DKA	+	Necrotising Fasciitis	*L. acidophilus*	+	*Candida*	N/A	Cured	[5]
2022	48	F	DKA	+	Necrotising Fasciitis	*L. salivarius* *L. iners*	+	*Candida. albicans*	9	Cured	This Case

LRINEC: Laboratory Risk Indicator for Necrotising Fasciitis; DKA: diabetic ketoacidosis; M: Male; F: Female. This table shows a list of soft-tissue infections associated with *Lactobacillus* spp. All patients were complicated by diabetes mellitus. The present case is the only one in which a mixed infection with *Lactobacillus* spp. was found.

*Lactobacillus* have been more frequently detected in the stool of patients with type 2 diabetes than in healthy individuals [13]. In addition, *Lactobacillus* including *L. iners* are endemic to the vagina of women [14]. In this case, the *Lactobacillus* may have entered the patient’s body via faeces and vaginal secretions through the folliculitis she had previously suffered, causing a mixed infection with *L. iners* and *L. salivarius*, resulting in NF. Although NF is generally more common in men [15], *Lactobacillus* spp. are part of vaginal microflora in women and maybe the causative organism of NF in women, especially those with diabetes.

NF caused by *Lactobacillus* has rarely been reported and its molecular and clinicopathological features are still unclear. Group A Streptococcus (GAS) secrete a variety of proteases that can destroy host tissues. They also express a variety of virulence factors that contribute to the severe tissue destruction characteristic of NF, including host cell adhesion, immune evasion, and tissue destruction by GAS-derived molecules. Although *S. aureus* has been shown to have similar characteristics, we were unable to identify any publications describing the molecular characteristics of NF caused by *Lactobacillus* [16]. In the two cases we found in the literature, the extent of necrosis was not described. The extent of NF is often difficult to determine from skin findings alone, especially in obese patients, because the infection progresses along the fascia [15]. The LRINEC score was useful for early diagnosis in this case. The LRINEC score is a clinical tool reported by Wong et al. [17] as a predictive diagnostic score for NF. The following six blood parameters are included in the score: CRP, total white blood cell count, haemoglobin, serum sodium, creatinine, and glucose. A score of ≥ 8 is considered as high risk factor for NF [17, 18]. Johnson et al. [19] reported that the sensitivity, specificity, positive predictive value, and negative predictive value of the LRINEC score in patients with diabetes were 100, 69, 16.6, and 100%, respectively. In the present case, the LRINEC score was 9, indicating that the patient was in a high-risk category and it also allowed for early diagnosis. Thus, the LRINEC score may be useful for the diagnosis of NF caused by low-virulence bacteria such as *Lactobacillus*.

The curative treatment for NF is early surgical intervention, and the prognosis is poor if surgery is delayed [20]. Although the timing of surgery was not described in the two case reports we retrieved, extensive necrosis due to *Lactobacillus* suggests that a delay in surgical intervention may be fatal. The present case suggests that early surgical intervention can be life-saving even in NF caused by bacteria with low virulence.

The prognosis of NF caused by *Lactobacillus* spp. is poorly understood. Regarding the mortality of NF caused by different types of bacteria, there was no difference between *Streptococci* and *Staphylococci*, on the other hand, there was a significant difference between *Vibrio* and *Aeromonas* in mortality [21]. In this case, the bacteria were detected using wound culture and the causative agent could be identified, although it was difficult to do so based on the course of the disease and skin findings alone. It should be noted that patients with compromised immune function can develop NF caused by *Lactobacillus spp.* However, due to the paucity of case reports, we cannot deduce definitive pathogenesis, specific features, or mortality of NF caused by *Lactobacillus* spp. The LRINEC score and early surgery may be helpful, but the limitations of the present case need to be evaluated with further case accumulation in the future.

In conclusion, morbidly obese, diabetic, and immunocompromised patients are at a risk of developing NF caused by less pathogenic bacterial species including *Lactobacillus*. In morbidly obese patients who are at risk, skin findings alone can delay the diagnosis of NF, but screening with the LRINEC score can be useful, and early surgical debridement life-saving. The clinical features and prognosis of NF caused by *Lactobacillus* spp. remain unknown, and further case reports are warranted.

## Data Availability

No datasets were generated or analysed during the current study.

## Abbreviations

BMI: body mass index
BUN: blood urea nitrogen
CRP: C-reactive protein
GCS: Glasgow Coma Scale
ICU: intensive care unit
LRINEC: Laboratory Risk Indicator for Necrotising Fasciitis
NF: necrotising fasciitis
MRSA: methicillin-resistant *Staphylococcus aureus*
NSAID: non-steroidal anti-inflammatory drug
NF: necrotising fasciitis

## References

[1] Stevens DL, Bryant AE (2017). Necrotizing soft-tissue infections. N Engl J Med.

[2] Peetermans M, de Prost N, Eckmann C, Norrby-Teglund A, Skrede S, De Waele JJ (2020). Necrotizing skin and soft-tissue infections in the intensive care unit. Clin Microbiol Infect.

[3] Salati SA (2022). Necrotizing fasciitis a review. Pol Przegl Chir.

[4] Anaya DA, Dellinger EP (2007). Necrotizing soft-tissue infection: diagnosis and management. Clin Infect Dis.

[5] Hubbard J, Jariwala B, Hill A, Gega A, Palesty JA (2018). A new bacterium, Lactobacillus acidophilus, causing necrotizing fasciitis. Am Surg.

[6] Cannon JP, Lee TA, Bolanos JT, Danziger LH (2005). Pathogenic relevance of Lactobacillus: a retrospective review of over 200 cases. Eur J Clin Microbiol Infect Dis.

[7] Aguirre M, Collins MD (1993). Lactic acid bacteria and human clinical infection. J Appl Bacteriol.

[8] Troche JMR, Adame EC, Díaz MÁV, Escudero OG, Chávez MAG, Chávez-Barrera JA (2020). *Lactobacillus acidophilus* LB: a useful pharmabiotic for the treatment of digestive disorders. Th Adv Gastroenterol.

[9] Kullar R, Goldstein EJC, Johnson S, McFarland LV. Lactobacillus bacteremia and probiotics: a review. Microorganisms. 2023;11(4). 10.3390/microorganisms11040896. 10.3390/microorganisms11040896 PMC10145752 37110319

[10] Franko B, Fournier P, Jouve T, Malvezzi P, Pelloux I, Brion JP (2017). Lactobacillus bacteremia: pathogen or prognostic marker?. Med Mal Infect.

[11] Fitzgerald JR, Foster TJ, Cox D (2006). The interaction of bacterial pathogens with platelets. Nat Rev Microbiol.

[12] Collins J, van Pijkeren JP, Svensson L, Claesson MJ, Sturme M, Li Y (2012). Fibrinogen-binding and platelet-aggregation activities of a Lactobacillus salivarius septicaemia isolate are mediated by a novel fibrinogen-binding protein. Mol Microbiol.

[13] Sato J, Kanazawa A, Ikeda F, Yoshihara T, Goto H, Abe H (2014). Gut dysbiosis and detection of live gut bacteria in blood of Japanese patients with type 2 diabetes. Diabetes Care.

[14] Zheng N, Guo R, Wang J, Zhou W, Ling Z (2021). Contribution of Lactobacillus iners to vaginal health and diseases: a systematic review. Front Cell Infect Microbiol.

[15] Wong CH, Wang YS (2005). The diagnosis of necrotizing fasciitis. Curr Opin Infect Dis.

[16] Olsen RJ, Musser JM (2010). Molecular pathogenesis of necrotizing fasciitis. Annu Rev Pathol.

[17] Wong CH, Khin LW, Heng KS, Tan KC, Low CO (2004). The LRINEC (Laboratory Risk Indicator for necrotizing fasciitis) score: a tool for distinguishing necrotizing fasciitis from other soft tissue infections. Crit Care Med.

[18] Bechar J, Sepehripour S, Hardwicke J, Filobbos G (2017). Laboratory risk indicator for necrotising fasciitis (LRINEC) score for the assessment of early necrotising fasciitis: a systematic review of the literature. Ann R Coll Surg Engl.

[19] Johnson LJ, Crisologo PA, Sivaganesan S, Caldwell CC, Henning J (2021). Evaluation of the Laboratory Risk Indicator for necrotizing fasciitis (LRINEC) score for detecting necrotizing soft tissue infections in patients with diabetes and lower extremity infection. Diabetes Res Clin Pract.

[20] Childers BJ, Potyondy LD, Nachreiner R, Rogers FR, Childers ER, Oberg KC (2002). Necrotizing fasciitis: a fourteen-year retrospective study of 163 consecutive patients. Am Surg.

[21] Al-Qurayshi Z, Nichols RL, Killackey MT, Kandil E (2020). Mortality risk in necrotizing fasciitis: national prevalence, trend, and burden. Surg Infect.

